# Pomegranate Peel Extracts as Safe Natural Treatments to Control Plant Diseases and Increase the Shelf-Life and Safety of Fresh Fruits and Vegetables

**DOI:** 10.3390/plants10030453

**Published:** 2021-02-27

**Authors:** Imen Belgacem, Maria G. Li Destri Nicosia, Sonia Pangallo, Ahmed Abdelfattah, Massimo Benuzzi, Giovanni E. Agosteo, Leonardo Schena

**Affiliations:** 1 Dipartimento di Agraria, Università Mediterranea, 89122 Reggio Calabria, Italy; imen.belgacem@unirc.it (I.B.); giulia.lidestri@unirc.it (M.G.L.D.N.); sonia.pangallo@unirc.it (S.P.); geagosteo@unirc.it (G.E.A.); 2 Institute of Environmental Biotechnology, Graz University of Technology, A-8010 Graz, Austria; ahmed.abdelfattah@tugraz.at; 3 BIOGARD, Division of CBC (Europe) srl, 24050 Bergamo, Italy; mbenuzzi@cbceurope.it

**Keywords:** pomegranate peel extracts, food by-products, polyphenols, punicalagins, plant pathogens, foodborne pathogens, coatings, shelf-life, antimicrobial activity, plant resistance

## Abstract

Although the Green Revolution was a milestone in agriculture, it was accompanied by intensive use of synthetic pesticides, which has raised serious concerns due to their impact on human and environmental health. This is increasingly stimulating the search for safer and more eco-friendly alternative means to control plant diseases and prevent food spoilage. Among the proposed alternatives, pomegranate peel extracts (PPEs) are very promising because of their high efficacy. In the present review, we discuss the complex mechanisms of action that include direct antimicrobial activity and induction of resistance in treated plant tissues and highlight the importance of PPE composition in determining their activity. The broad spectrum of activity, wide range of application and high efficiency of PPEs against bacterial, fungal and viral plant pathogens suggest a potential market not only restricted to organic production but also integrated farming systems. Considering that PPEs are non-chemical by-products of the pomegranate industry, they are perceived as safe by the public and may be integrated in circular economy strategies. This will likely encourage agro-pharmaceutical industries to develop commercial formulations and speed up the costly process of registration.

## 1. Introduction

Starting from the Green Revolution, the development of modern and high yielding crop varieties has led to intensive monoculture cropping systems, loss of biodiversity and more susceptibility to diseases [1,2]. Together with climate change and market globalization, the emergence and spread of resistant pathogens has become a serious concern [2,3]. For decades, the Green Revolution has been accompanied by the intensive use of chemicals which reduces agricultural production stability and sustainability [2,4]. Chemical pesticides pose serious risks to human and environmental health, with negative impacts on non-target microorganisms and increasing selection for antimicrobial resistant strains [5,6]. With the latest European legislative restrictions and the rise of consumer awareness in food safety and healthy living, the development of safe and environmentally friendly alternative control means to control plant diseases has become an imperative need [7,8,9]. Several alternative methods have been proposed, including plant extracts, which are usually applied alone or as a part of integrated pest management programs [10,11,12,13]. In this regard, extracts from pomegranate peel have emerged as a source of very promising antimicrobial substances to control plant and foodborne pathogens. Pomegranate peels have been used in folk medicine since ancient times because of their health benefits due to the presence of various useful compounds [14]. A number of scientific evidences have proved the therapeutic and antioxidant activity of pomegranate peel extracts (PPEs) against many critical maladies including cancer, inflammation, diabetes, cardiovascular diseases, etc. [15,16,17,18].

**Figure 1:**
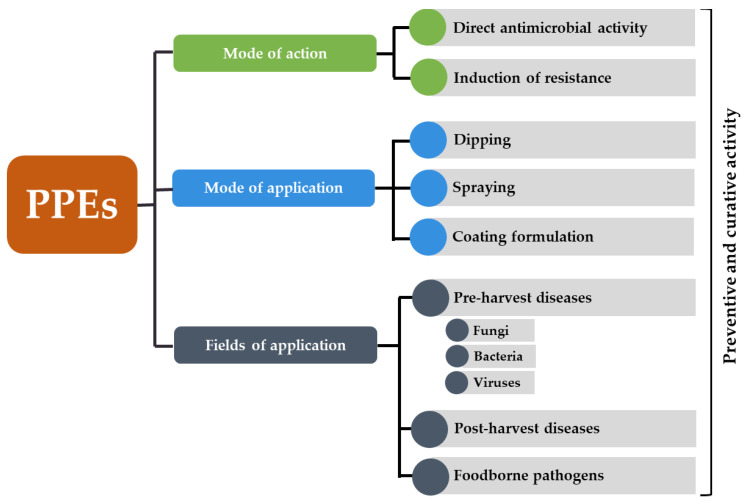
Schematic representation of fields of application, mode of application and mode of action of pomegranate peel extracts (PPEs) to control plant diseases and increase the shelf-life and safety of fresh fruits and vegetables.

This review focuses on PPEs as natural preparations to control plant and foodborne pathogens associated with fresh fruits and vegetables. The main active compounds responsible for PPEs biological activity, the mechanism of action, the range of activity and the potential practical applications are discussed (Figure 1). Being natural products, PPEs are likely to receive a great deal of attention and acceptance from the consumer who increasingly does not positively perceive chemical pesticides and food additives [19,20]. Furthermore, the valorization of by-products such as pomegranate peel is an important shift towards more sustainable food and agricultural systems.

## 2. Bioactive Components of Pomegranate Fruit

Pomegranate (*Punica granatum* L., Punicaceae) is an ancient fruit, widely used in traditional medicine for its protective and therapeutic effects. The main bioactive components in pomegranates are ellagitannins, mainly represented by punicalagin, a type of phenolic compound typical of pomegranate and few other plant species [21]. Ellagitannins are hydrolysable tannins known to be potent free radical scavengers with many nutraceutical effects such as antioxidant, anti-inflammatory, anti-microbial and anti-carcinogenic properties, etc. [22,23,24]. However, different studies focusing on the isolation and characterization of the active components of pomegranate have shown that although punicalagins are the most important constituent, the high biological value of the fruit is determined by the chemical synergetic action of the total fruit phytoconstituents rather than by a single component [22,25,26]. These findings have pushed researchers to consider the whole extract rather than punicalagins alone.

Phytochemical screening of different parts of the pomegranate fruit including peel, arils and seeds revealed a high predominance of polyphenols in the peel part [26,27]. This explains the particular use of the peel in folk medicine [14]. Since the peel of pomegranate accounts for about 50% of the total fruit weight, it represents a rich source of bioactive components. In particular, punicalagins and gallic acids are the main active components in the peel and have been correlated with the antimicrobial activity of the extract [28,29,30]. However, an accurate standardization of PPE preparations is needed since the polyphenolic content may greatly vary according to several factors. For instance, the solvent choice was shown to have a significant impact on the concentration of polyphenols [31,32,33,34,35,36]. Al-Zoreky [37] showed that an 80% methanolic extract was richer in polyphenols compared to hot water and diethyl ether extracts and, therefore, exerted a higher antimicrobial activity against *Listeria monocytogenes*, *Staphylococcus aureus*, *Escherichia coli* and *Yersinia enterocolitica*. Similarly, Tayel et al. [38] found that a methanolic peel extract was more effective than ethanol and water extracts in controlling *Penicillium digitatum*. On the other hand, Romeo et al. [32] found a higher concentration of anthocyanins and phenols in an ethanolic extract compared to other extracts. They also suggested that the use of a safe chemical (food grade ethanol) for extractions is important to obtain eco-friendly and safe antimicrobial preparations.

Similar to the extraction method, factors such as maturity stage, variety, growth region and environmental conditions can have a significant impact on the polyphenolic composition and on the antimicrobial activity of pomegranate extracts. For instance, Glazer et al. [30] reported different levels of punicalagins in peel extracts from different pomegranate accessions. More recently, the importance of pomegranate cultivars was confirmed since chemical composition of extracts greatly varied according to genotypes and significantly affected the antifungal activity [39].

## 3. Mechanisms of Action

The mechanisms by which the bioactive components of PPEs exert their activity have not been completely elucidated, although a consistent quantity of information is now available suggesting both a direct antimicrobial activity and the activation of resistance responses in treated plant tissues. The direct antimicrobial activity is one of the most investigated features of PPEs, although most studies have focused on human-associated microorganisms [35,36,40]. In vitro trials showed strong inhibitory activity against the germination of conidia and the mycelium growth of major plant fungal pathogens including *Botrytis cinerea*, *Penicillium digitatum*, *Penicillium expansum*, *Penicillium italicum*, *Alternaria alternata*, *Stemphylium botryosum*, *Colletotrichum acutatum sensu stricto, Fusarium oxysporum*, *Aspergillus parasiticus*, *Monilinia laxa* and *Monilinia fructigena* [21,28,29,30,37,40,41,42]. The level of antifungal activity can greatly vary according to extract type and pathogen species. For instance, an ethanolic PPE completely inhibited the germination of conidia of *B. cinerea* and *C. acutatum*, while it was less effective against *P. digitatum* and *P. expansum* which were reduced by 91.0% and 82.7%, respectively [41,42]. An aqueous PPE inhibited the mycelial growth of *A. alternata*, *S. botryosum* and *Fusarium* spp. but it was ineffective against *P. expansum*, *P. digitatum* and *B. cinerea* [30].

Additionally, since PPEs combine a direct antifungal activity with the inhibition of the toxin biosynthesis, they can be used against mycotoxigenic fungi. In a recent study, a methanolic PPE significantly delayed conidial germination and hyphal elongation rate of *Aspergillus flavus* and *Fusarium proliferatum*. Furthermore, the production of aflatoxins was reduced by 97% using the extract alone, and it was completely inhibited combining the PPE with the azole fungicide prochloraz (PRZ) [43].

Furthermore, PPEs have also been reported to exert a high antimicrobial activity against both Gram-positive and negative bacteria [35,40,44,45,46]. They proved effective against important plant pathogens such as *Clavibacter michiganensis* subsp. *michiganensis*, *Pseudomonas syringae* pv. *actinidiae*, *Pseudomonas syringae* pv. *syringae*, *Erwinia carotovora* and *Xanthomonas campestris* [47,48] and against food-borne pathogens such as *Salmonella* spp. and *L. monocytogenes* [36,49]. Belgacem et al. [49] showed strong and quick bactericidal and bacteriostatic activity against *L. monocytogenes*. Strong activity against bacteria was also confirmed by analyzing the epiphytic population of olive drupes and citrus fruits after PPE treatments [41,50].

Numerous studies correlated the antifungal and antibacterial activity of PPEs to their high concentration of polyphenols, particularly punicalagins and ellagic acids. Rongai et al. [38] found that punicalagins are responsible for the inhibition of the mycelial growth of *Fusarium oxysporum* f. sp. *lycopersici* and highlighted that PPEs are among the most effective plant extracts in preventing the germination of *F. oxysporum*. Similar results were reported for the conidial germination and hyphal growth of the mycotoxigenic fungi *A. flavus* and *F. proliferatum* [43]. Microscopic observation of *Fusarium sambucinum* mycelium treated with methanol PPE revealed hyphal morphological modifications including curling, twisting, and collapsing [51]. Cell empty cavities and disintegration of cytoplasmic organelles were also observed. Similarly, an abnormal mycelia structure of *M. laxa* and *M. fructigena* was recorded following a PPE treatment [52]. Furthermore, analysis of the sterol composition of *A. flavus* showed potential inhibition by PPE of the ergosterol biosynthesis, a pathway responsible for fungal cell membrane fluidity and permeability and required for hyphal elongation. Akhtar et al. [24] and Foss et al. [53] reported that PPE polyphenolic compounds combine with proteins of the fungal cell membrane and cause the cell death by increasing permeability. Furthermore, PPEs can decrease the pH gradient around the cell membrane and cause the cell death by increasing permeability [29,44]. On the other hand, Wu and Kim [54] and Dey et al. [55] highlighted that the reaction of polyphenols with sulfhydryl groups may induce enzymatic inhibition and microbial starvation. In this regard, Sudharsan et al. [43] observed that PPEs can inhibit aflatoxin production in *A. flavus* by inhibiting specific enzymes in the pathway of aflatoxin biosynthesis. In addition, a specific study on the proteomic effects of punicalagin on *S. aureus* showed that it adversely alters bacterial growth by disrupting iron homeostasis and inducing SOS responses, possibly through DNA biosynthesis inhibition [56]. Although some reports suggested that the presence of the outer lipopolysaccharide membrane in Gram-negative bacteria could reduce the ability of PPEs to alter and affect cells, other investigations have shown a high efficacy against Gram-negative bacteria such as *Salmonella* Enteritidis [36].

Besides the direct antimicrobial activity, there is clear evidence showing that PPEs induce resistance in plant tissues. Pangallo et al. [41,57] indirectly demonstrated that PPE could activate the plant defense responses in olive drupes inoculated with *C. acutatum* and in citrus fruits inoculated with *P. digitatum* and *P. italicum* by observing a reduction in disease incidence without a direct contact between the pathogens and the extract. Furthermore, on grapefruit, an increase in reactive oxygen species (ROS) was detected following the PPE treatment, reaching a peak after 24 h post-treatment [57]. The same study revealed the activation of several genes involved in plant defense responses such as CHI, CHS, MAPK, MAPKK and PAL. More recently, a transcriptomic analyses of citrus fruit revealed the activation of many genes and pathways involved in plant defense responses [58]. Particularly, the study showed the induction of nine enzymes implicated in the biosynthesis of antibiotics. The authors suggested that the induction of this pathway might be one of the main mechanisms of action of PPE to counteract microbial infections and correlated the activation of these enzymes with the extract composition since polyphenols are proved to induce resistance in plant tissues [59].

The activation of resistance responses may explain the observed long persistence of efficacy after PPEs treatments [42,50]. However, specific investigations are needed to determine the persistence of host resistance responses after PPE treatment and their degradation rate within the host tissues.

### Preventive and Curative Actions

An important feature of PPEs is their high efficacy in both preventive and curative treatments [42,51,57]. The control of already established infections is important since most of the alternative control means are only effective when applied before the infection takes place. On olives artificially inoculated with *C. acutatum sensu stricto*, PPE treatments made 6, 12 and 24 h after inoculations significantly reduced the incidence of rots suggesting the possible control of already established infections. Similar findings were obtained on apples inoculated with *P. expansum* and on grapefruits and lemons inoculated with *P. digitatum* and *P. italicum* [42]. Furthermore, a strong curative action was also confirmed in field trials to control anthracnose in olive orchards characterized by a high incidence of latent infections [41]. Authors speculated on the importance of this feature since latent infections play a fundamental role in the epidemiology of olive anthracnose [60,61]. These results also suggested the potential use of PPEs to increase the shelf-life of fresh fruits and vegetables since treatments applied just before or soon after harvest could be used to reduce latent infections and protect fruits during harvesting, packaging and storage [37,62].

The exact mechanisms by which PPE exerts its curative action is not completely understood. The ability of the extract to rapidly activate resistance response in plants and induce the production of antifungal compounds is likely to play a major role [58]. Additionally, a direct antifungal activity of the extract against the colonizing fungi might be possible, but currently there is no evidence regarding the penetration and diffusion of PPE active components into the host tissues.

## 4. Practical Applications

### 4.1. Post-Harvest Diseases

The control of postharvest diseases and the extension of the shelf-life of fresh fruits and vegetables are the most investigated fields of application of PPEs (Table 1). Fresh fruits and vegetables are very perishable and their quality can quickly deteriorate due to the high respiratory metabolism, biosynthesis and action of ethylene, transpiration and decay which is mainly caused by fungi [13]. Postharvest decay can be caused by latent and quiescent infections established in the field between flowering and fruit maturity and by wound infections that occur during harvesting and subsequent handling and storage [63]. Currently, synthetic fungicides are still used to control postharvest diseases; however, the growing health and environmental concerns over pesticide disposal and residue levels have led to more and more stringent regulations that withdraw most postharvest fungicides. For instance, in many European countries the postharvest use of fungicides is completely prohibited or limited to just a few chemicals registered on specific commodities. Furthermore, the few currently authorized chemicals are increasingly threatened by the development of pathogen resistant strains.

Many alternative control means have been proposed in the last 30 years to replace chemical fungicides. However, it is generally accepted that multiple interventions with different methods are required at different stages of the disease cycle to achieve acceptable levels of protection [9]. The reduction of losses is very important for harvested commodities considering their high value and the consequent economic impact of postharvest rots. In this context, PPEs appear particularly promising since they demonstrated a high level of efficacy on a broad range of postharvest diseases (Table 1). Furthermore, based on their complex mechanism of action, long persistence of activity and both preventive and curative actions, they guaranty high level of protection and flexibility in terms of method of application [50]. The lack of reliability is a major limitation of other alternative control methods, including those based on microbial antagonists, as they are greatly influenced by external factors such as environmental conditions and may produce inconsistent results over the time [64]. Methanolic PPEs proved highly effective against the postharvest dry rot of potato tubers caused by *F. sambucinum* and postharvest citrus rots caused by *P. digitatum* [37,51]. Similarly, Rongai et al. [29] reported about the use of an aqueous PPE to effectively control grey mold and extend the fruit shelf-life of strawberries. An ethanolic PPE showed a wide spectrum of activity against several postharvest diseases including gray mold on table grapes and sweet cherries, brown rot on sweet cherries, green and blue mold on citrus and blue mold on apples [41,42,57]. Under large-scale trials simulating commercial conditions, this extract almost completely inhibited citrus postharvest rots and proved more effective than Imazalil (IMZ), a fungicide commonly used for postharvest treatments. Interestingly, high levels of protection were achieved with both pre- and post-harvest applications of PPE, confirming a high level of flexibility in terms of both time and method of application [50]. PPE field treatments may be particularly useful for delicate fruit species such as strawberries and table grapes that cannot be subjected to water dip treatments after harvest [65]. Furthermore, these treatments may be strategic to protect fruits from wound infections occurring during harvesting and pre-packaging phases, particularly in less technologically advanced countries where treatments are performed with simple equipment used for conventional fungicide spraying. On the other hand, postharvest dipping treatments can be easily and cheaply integrated into common packinghouse operations by adding the active ingredient to the water used to wash fruit and/or reduce temperature (hydro-cooling).

**Table 1:** Overview of different applications of PPEs to control plant diseases and foodborne pathogens.

Field of Application	Pathogens	Host	References
Pre-harvest diseases	*Fusarium oxysporum*	Tomato	[28]
	*Colletotrichum acutatum*	Olive	[41]
	*Uncinula necator*	Grape	[66]
	*Pseudomonas syringae* pv*. tomato*	Tomato	[67]
	*Xylella fastidiosa*	Olive	[68]
	Tomato Spotted Wilt Virus	Tobacco, carnation	[66]
Post-harvest diseases	*Fusarium sambucinum*	Potato tubers	[51]
	*Botrytis cinerea*	Lemon, strawberry, grape	[29,42,69]
	*Monilinia laxa*	Sweet cherries, apple	[42]
	*Monilinia fructigena*	Apple	[52]
	*Penicillium digitatum*	Lemon, grapefruit, orange	[37,42,57,70]
	*Penicillium italicum*	Lemon, grapefruit	[42,57]
	*Penicillium expansum*	Apple	[42]
	*Colletotrichum gloeosporioides*	Capsicum	[71]
	*Colletotrichum acutatum*	Olive	[41]
Foodborne pathogens	*Listeria monocytogenes*	In vitro and in vivo (pear, apple, melon)	[36,49,72,73,74]
	*Salmonella spp.*	In vitro	[35,36]
	*Escherichia coli*	In vitro	[35,36,72,73]
	*Staphylococcus aureus*	In vitro	[35,36,72,75]
	*Clostridia*	In vitro	[76]
	*Yersinia enterocolitica*	In vitro	[36]
	*Bacillus subtilis*	In vitro	[35,72]
	*Bacillus cereus*	In vitro	[72]
	*Vibrio parahaemolyticus*	In vitro	[74]

Positive results have also been achieved by adding PPEs to edible coatings. The addition of PPE to chitosan and locust bean gum coating significantly reduced disease incidence of green mold on oranges artificially inoculated with *P. digitatum* [77]. Similarly, the addition of the extract to a polysaccharide-based edible coating extended the shelf-life of *Capsicum annuum* L. [71]. Authors reported an interesting antifungal activity against *Colletotrichum gloeosporioides* and a significant retention of physiological loss in weight, firmness, color and ascorbic acid, which enabled the conservation of sensory scores and the extension of the shelf-life. Similarly, pectin-based edible coatings formulated with PPE were used to extend the shelf-life of fresh-cut persimmon [78]. Finally, a formulation containing cassava starch, chitosan, essential oil and pomegranate peel extract was utilized to preserve tomatoes stored at room temperature [77].

The high antimicrobial activity of PPEs also suggests their potential use as natural preparations for the sanitation of water, environment and containers in the post-harvest industry. The aerosolization of a methanolic PPE proved effective for the sanitation of trailer cabinets and containers used for the transportation and storage of citrus fruit [37]. Similarly, another ethanolic extract was proposed as a safe means to reduce the microbial contamination in recirculated water and avoid the use of chlorine or other sanitizers commonly used in citrus packinghouses [50].

### 4.2. Pre-Harvest Diseases

#### 4.2.1. Control of Fungal Field Diseases

Currently, there are few reports on the use of PPEs to control field fungal diseases; however, the high efficacy and broad range of antifungal activity demonstrated by different PPEs in in vitro and laboratory experiments encouraged scientists to further investigate this field of application (Table 1). Indeed, available field data suggest the possible use of PPEs to control a broad range of diseases caused by necrotrophic, hemibiotrophic and biotrophic fungal pathogens. For instance, the incorporation of a PPE in soils artificially inoculated with *F. oxysporum* f. sp. *lycopersici* significantly reduced the population of the pathogen and increased the number of healthy tomato plants [28,69]. Results showed high efficiency of the extract, similar to the standard fungicide dicloran (Marisan 50 PB). In another study, the application of a methanolic PPE as seed or soil treatment significantly deceased pre- and post-emergence damping off of tomato caused by *F. oxysporum* under greenhouse conditions [79]. Soil treatment was more effective than seedling treatment. It is worth mentioning that it was reported that a high concentration of the extract may induce allelopathic activity in tomato plants [28].

Regarding hemibiotrophic fungi, field trials conducted in commercial olive orchards to control olive anthracnose demonstrated a very high efficacy of an ethanolic PPE that proved significantly more effective than copper, traditionally used to control this disease [41]. In particular, the application of the extract in the early ascending phase of the disease outbreak completely inhibited the development of natural rots. Authors speculated that since their extract is obtained using safe chemicals with no apparent phytotoxic effect on treated olive fruit, it may be regarded as a safe and effective natural antifungal preparation.

**Figure 2:**
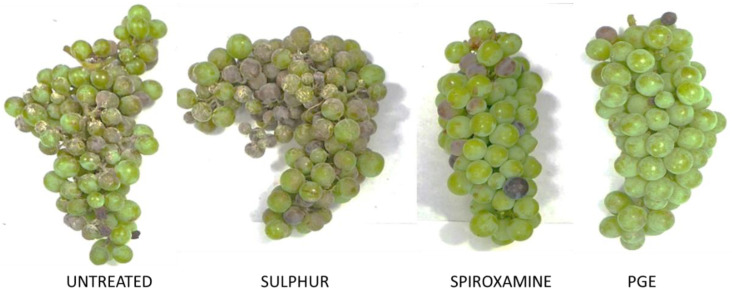
Representative results of trials conducted on grapevine (cv. Aglianico) to control the powdery mold fungus *Uncinula necator*. Treatments were made on 8 July 2016 with wettable sulphur (TIOVIT^®^) at 2 g/l, Spiroxamine (PROSPER^®^) at 0.8 mL/l and PGE at 6 g/l. Untreated grapes were used as control. Photos were made at the beginning of the grape veraison, on 17 August 2016.

Recently, the use of an ethanolic PPE to control the grape powdery mold fungus *Uncinula necator* has been patented, highlighting the possible use of PPEs also against biotrophic pathogens (Figure 2) [66]. On grapevine cv. Aglianico, three treatments at intervals of 15 days, during the phenological phases of fruit set, pre-bunch closure and bunch closure, reduced the disease incidence by 71%, reaching an efficacy equal to that of the systemic fungicide Spiroxamine (Prosper^®^, Bayer Crop Science Italy).

The use of PPEs to control fungal field diseases is very intriguing particularly because they may significantly contribute to reduce or replace the use of synthetic fungicides in agriculture. In fact, the broad spectrum of activity and the high level of efficacy suggest a potential market not restricted to organic productions since it may also include conventional and/or integrated farming systems.

#### 4.2.2. Control of Bacterial Field Diseases

The possible use of PPEs to control plant bacterial diseases is particularly interesting since copper is the only authorized effective bactericide in most countries and frequently does not provide an accurate level of protection. Copper treatments are protectants without curative or systemic action and, therefore, must be applied prior to infection. Furthermore, copper can have serious ecological drawbacks. It accumulates in the soil and toxifies the natural microbial population and fauna. It may also have harmful effects on humans. This led to its insertion in the list of substances identified as “candidates for substitution” by the European Commission under Regulation (EC) No 1107/2009.

The use of PPEs to control plant bacteria was first proposed in 2013 [67]. Authors reported a good efficacy against the tomato bacterial speck caused by *Pseudomonas syringae* pv. *tomato* and speculated on the importance of their findings considering the lack of valid alternative compounds and the non-availability of commercial resistant cultivars of tomato. More recently, a hydroalcoholic PPE was utilized to treat olive trees affected by *Xylella fastidiosa*, a systemic bacterium that colonizes the xylem tissues. Experiments carried out over a four-year period (2016–2019) showed a general improvement of the plant’s health after trunk injections [68,80]. The induction of resistance in plant host tissues treated with PPEs and the broad range of antibacterial activity demonstrated in in vitro studies suggest the potential use of PPEs as safe control means in other plant bacterial pathosystems.

#### 4.2.3. Control of Viral Field Diseases

An Italian patent has recently covered the use of an alcoholic extract of pomegranate peel to control plant viruses [66]. The artificial inoculations of the extract on tobacco and carnation plantlets prevented the infection of tomato spotted wilt virus (TSWV). According to the authors, the extract activates specific resistance responses in the plant and prevents viral infections. Although further investigations are needed to confirm the possible implementation of PPEs in plant virus control strategies, these preliminary results are highly important. In fact, plant viruses are challenging pathogens hard to control and only in rare cases can be controlled through the application of pesticides or other chemicals [81]. Therefore, rigorous research should be dedicated to investigate the application of PPEs to control plant virus diseases.

## 5. Foodborne Pathogens Associated with Fresh Fruits and Vegetables

Much effort is devoted to plant associated pathogens as they affect the health and productivity of many important crops. However, some of these pathogens directly infect also human beings, resulting in cross-kingdom pathogenicity [82,83]. The risk of human borne pathogens is very high, leading to human illness and in some cases to death. Despite the wide range of preservation techniques, food borne diseases remain an important global health problem. Serious food-borne outbreaks and food recalls were recorded on fresh produce resulting in acute food poisoning and huge economic losses [84]. This is mainly attributed to many factors, including cross contamination, mechanical wounding and especially the development of antibiotic resistant foodborne pathogens which has been an emerging public-health threat [83,85]. As processed foods are very susceptible to physical and biological deterioration, the need to develop natural preservation techniques is highly required from the consumer who is moving toward a healthier diet. In this regard, PPEs were reported to exert strong bactericidal and bacteriostatic activity against several Gram-positive and negative foodborne bacteria including *Salmonella* spp., *L. monocytogenes, E. coli*, *Pseudomonas aeruginosa, Clostridia*, *S. aureus*, *Y. enterocolitica*, *Bacillus subtilis*, *Bacillus cereus* and *Vibrio parahaemolyticus* (Table 1) [24,36,72,74,86]. It has been demonstrated that PPEs can extend the shelf-life and maintain the microbiological, chemical and sensorial quality of food products when applied individually or in combination with other antimicrobial agents. For instance, a strong activity of PPE against *L. monocytogenes* was shown on fresh-cut pear, melon and apple fruits, suggesting its possible implementation in the industry of ready-to-eat fresh-cut products to guarantee high levels of control and safety [49]. Interestingly, PPEs showed synergetic action with several other bioactive agents including chitosan, alginate, biocontrol agents and other plant extracts [70,71,78,87,88]. Furthermore, the possible use of PPEs in edible coating formulations is particularly interesting in light of the rapidly increasing interest of food industries for new active packaging materials [89,90]. For instance, the incorporation of a PPE in gelatine film-forming solution improved the antioxidant properties and antimicrobial activity of the active packaging against *S. aureus*, *L. monocytogenes* and *E. coli* [75]. Similarly, the incorporating of PPE and sodium dehydroacetate in a PVA film increased the antioxidant activity of the film and the bacteriostatic effect against *E. coli* and *S. aureus* [91]. Recently, PPE immobilized electrospun active nanofibers were proposed as an excellent food wrapping material to preserve the sensory properties and extend the shelf-life of meat and other food product [92]. Currently, many industries are interested in introducing PPEs in their food products as a functional ingredient because of their excellent antioxidant, anti-inflammatory and antibacterial effects, and their wide range of application which is not restricted to fresh fruits and vegetables but also many other food products including meat, sausages, fish, bread, juice, etc.

Interestingly, research showed that the application of PPEs on food products is not only useful for their antimicrobial activity against foodborne pathogens but also for their beneficial effect in stimulating the growth of human gut microbiota. In fact, one key finding on PPEs is their potential prebiotic effect, mainly due to their richness in polyphenols, particularly ellagitannins, which are reported to selectively modulate the growth of susceptible microorganisms [76,93,94,95]. It has been reported that PPEs stimulate the growth of *Bifidobacterium* and *Lactobacillus* strains, some of the most important taxa involved in food microbiology and human nutrition [94,96]. Neyrinck et al. [95] found that PPE combined with the probiotic *Lactobacillus rhamnosus* significantly reduces lipid accumulation, suggesting its potential implementation in obesity prevention diets.

## 6. Conclusions

Although the use of natural treatments to control plant diseases and foodborne pathogens associated with fruit and vegetable is gaining great interest from the scientific community as well as consumers, many of the investigated methods are associated with major limitations including the lack of curative or preventive effects, impersistent efficiency, risk of fruit injury and incompatibility with other treatments [97]. In this regard, PPEs are proving to be a viable and versatile natural alternative with high efficacy and a broad range of activity. However, even though no signs of phytotoxicity have been reported up to now, further research is needed to confirm the safety of PPEs for plants, human health and the environment. In this context, the already documented therapeutic action of PPEs would catch the attention and acceptance of the public and encourage the pharmaceutical industry to investigate these aspects and speed up the costly process for the registration as a natural antimicrobial and fungicide. Furthermore, the wide availability of pomegranate peel as a by-product of processing factories should contribute to obtaining low-cost formulations able to compete with traditional chemical compounds.
